# Malignant Ependymoblastoma Mimicking a Benign Pilocytic Astrocytoma

**DOI:** 10.3390/neurolint12030010

**Published:** 2020-10-30

**Authors:** Pham Minh Thong, Nguyen Minh Duc

**Affiliations:** 1 Department of Radiology, Hanoi Medical University, Ha Noi 100000, Vietnam; phamminhthong@hmu.edu.vn or; 2 Department of Radiology, Pham Ngoc Thach University of Medicine, Ho Chi Minh City 700000, Vietnam; 3 Department of Radiology, Children’s Hospital 02, Ho Chi Minh City 700000, Vietnam

**Keywords:** ependymoblastoma, pilocytic astrocytoma, fourth ventricle tumor, children

## Abstract

Ependymoblastoma is an uncommon, exceedingly malignant brain neoplasm that adversely influences children’s quality of life. Ependymoblastoma represents a subtype of primitive neuroectodermal tumors, categorized as grade IV, according to the 2007 World Health Organization (WHO) classification of central nervous system tumors. Ependymoblastomas are often located in the supratentorial zone and often associated with the ventricular system. Histopathological sections of the tumor revealed uniform, primitive, small blue cells, with multi-layered rosettes, accompanied by abundant mitoses. The clinical and imaging features of ependymoblastomas are not specific, which can result in misdiagnosis as other brain neoplasms. In this paper, we described the identification of a fourth-ventricular ependymoblastoma that was misdiagnosed as pilocytic astrocytoma, despite the utilization of advanced magnetic resonance imaging (MRI) protocols.

## 1. Introduction

Ependymoblastoma was initially defined by Bailey and Cushing, who observed that ependymal-derived tumors can be classified into two subgroups: ependymomas and ependymoblastomas [1]. However, this subclassification was controversially rejected, because Bailey and Cushing later agreed that their initial architectural discrimination criteria were insufficient and inappropriately associated with histopathological behavior. In 1970, Rubinstein effectively demonstrated that ependymoblastoma, a specific form of embryonic central nervous system (CNS) neoplasm, can be distinguished as a strongly cellular neuroectodermal tumor, combined with the appearance of multiple, real, ependymoblastic rosettes [2]. The 2007 World Health Organization (WHO) classification guidelines for CNS tumors defined ependymoblastoma as a primitive neuroectodermal tumor (PNET), with the capacity to differentiate extensively from ependymal cells [3].

Ependymoblastomas present primarily in children [4,5,6,7,8,9]. The majority of ependymoblastomas are positioned supratentorially, followed by infratentorially [7,8,9]. Rarely, ependymoblastomas are found outside of the CNS, such as the sacrococcygeal region [10], rectovaginal space [11], or ovaries [12]. In this paper, we described one atypical case of infratentorial ependymoblastoma, which was misdiagnosed as pilocytic astrocytoma, despite the extensive use of innovative magnetic resonance imaging (MRI) sequences, such as diffusion-weighted imaging (DWI), spectroscopy, and T1-perfusion. 

## 2. Case Presentation

This study was approved by the Institutional review board of Children’s Hospital 2 (Ref: 352/NĐ2-CĐT dated 13 March 2020). Written informed consent from the patient’s legal guardian was obtained for the publication of this case report and any accompanying images.

**Figure 1:**
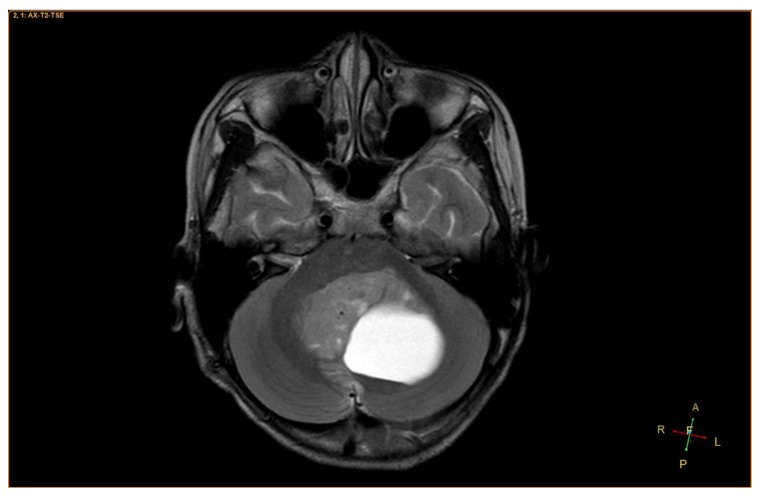
A heterogeneous mass, located in the fourth ventricle, containing both solid and cystic components, on axial T2-weighted imaging.

**Figure 2:**
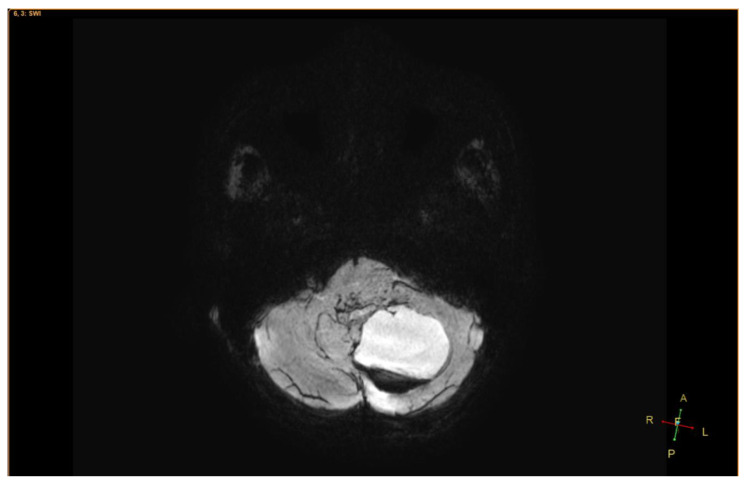
On susceptibility-weighted imaging, a hemorrhage in the cystic fluid was clearly observed.

**Figure 3:**
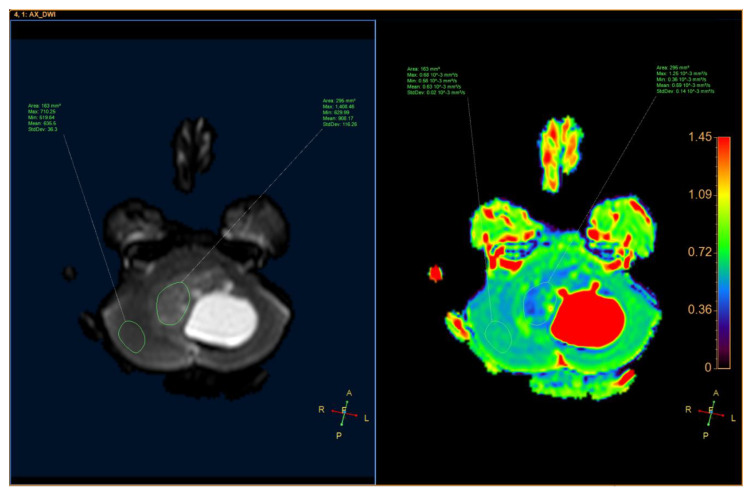
Axial apparent diffusion coefficient of the solid component of the mass and the parenchyma.

**Figure 4:**
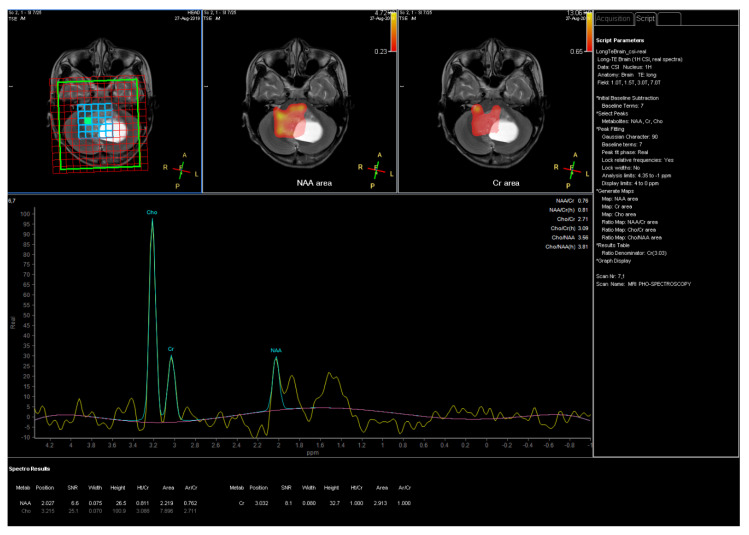
Magnetic resonance imaging (MRI) spectroscopy for the solid component of the mass.

**Figure 5:**
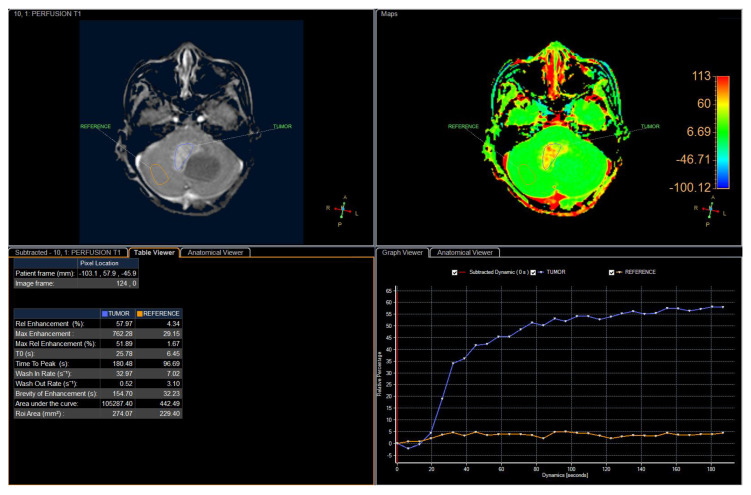
The detailed perfusion parameters for the solid component of the mass and the parenchyma.

**Figure 6:**
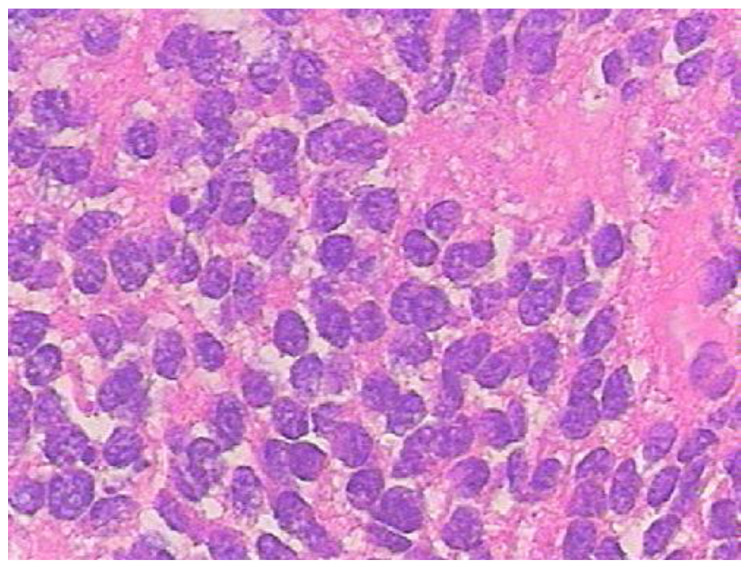
Histopathological findings showed unclassical, multilayered, ependymoblastic rosettes, with densely packed, small, blue cells accompanied by nuclear pleomorphism and strong mitotic activities (H&E staining, × 400).

An 11-year-old female, suffering from headache, nausea, and vomiting for 6 months, was admitted to the Department of Neurosurgery, Children’s Hospital 2. Her medical profile manifested no abnormalities, and no neurological deficits were detected during clinical evaluation. The routine laboratory test results and tumor markers were within normal ranges. Brain MRI, with contrast agent, was immediately indicated. The MRI findings showed no signs of hydrocephalus signs and no cerebral lesions. However, on T2-weighted imaging, a heterogeneous mass was observed in the fourth ventricle, with both solid and cystic appearance (56 mm × 48 mm × 51 mm) and without surrounding cerebellar vasogenic edema (Figure 1). Hemosiderin deposition, due to an old hemorrhage, was observed within the cystic fluid component, on susceptibility-weighted imaging (Figure 2). The mean apparent diffusion coefficient (ADC) values for the parenchyma and the solid component of the mass were 0.63 and 0.59 × 10^−3^ mm^2^/s, respectively (Figure 3). On MRI spectroscopy, the choline/N-acetyl aspartate ratio of the solid component of the mass was 3.56 (Figure 4). The relative enhancement (%), peak enhancement, peak relative enhancement (%), time to peak (s), wash-in rate (s^−1^), wash-out rate (s^−1^), and area under the curve values for the parenchyma compared with the solid mass component, as measured using the axial T1-perfusion map, were 4.34 vs. 57.97, 29.15 vs. 762.28, 1.67 vs. 51.89, 96.69 vs. 180.48, 7.02 vs. 32.97, 3.10 vs. 0.52, and 442.49 vs. 105,287.40, respectively (Figure 5). The first diagnosis, which relied on available clinical and subclinical information, was pilocytic astrocytoma. The patient underwent gross-total tumor resection. The histopathological evaluation of the excised tumor tissues revealed the tumor to be an ependymoblastoma (Figure 6). Two weeks later, the patient was released and received adjuvant chemo- and radiotherapy at a distinct oncological center.

## 3. Discussion

Ependymoblastoma, which most commonly adversely affects children, is an extraordinarily unusual and profoundly malignant CNS tumor. According to the 2007 WHO classification guidelines for CNS tumors [3], ependymoblastoma represents an embryonal, grade IV tumor, which was originally listed as a subtype of PNETs before being categorized as an embryonal tumor with multilayered rosettes (ETMR) in the 2016 update of WHO classification guidelines for CNS tumors [13]. Diagnosis depends on clinical characteristics, imaging features, and therapeutic methods, which are not standardized for ependymoblastoma due to the rarity of case reports and relative lack of original studies; therefore, most neuroradiologists and neurosurgeons are unfamiliar with ependymoblastomas [7,8,9]. The prognosis for ependymoblastomas in both children and adults is exceedingly dismal [1,2,3,4,5,6,7,8,9]. Histopathologically, the tumor features uniform neuroectodermal cells and ependymoblastic rosettes, coupled with highly mitotic activities, which can be discriminated from ependymal rosettes [2,3,13]. In this article, we reported the unusual appearance of a malignant ependymoblastoma, which mimicked a benign, pilocytic astrocytoma.

Ependymoblastomas are usually described as large, well-defined masses (diameter^max^ ≥ 5 cm), without surrounding vasogenic edematous parenchyma [8,9]. Among reported ependymoblastomas, 77% showed hemorrhage inside the tumor [8,9]. Approximately 18% of ependymoblastomas present as large cysts [8,9]. Ependymoblastomas are usually associated with ventricular systems [7,8,9]. Ependymoblastomas are believed to originate from ectopic neuroepithelial precursor cells, which are often situated in the periventricular white matter [5,14].

The ependymoblastoma identified in this report appeared as a large, heterogeneous, cystic, and solid mass, located in the fourth ventricle, with the strong enhancement of the solid portion and non-enhancement of the cystic element, which is a very typical hallmark of pilocytic astrocytoma. Although innovative MRI sequences were utilized, including DWI, MRI spectroscopy, and T1-perfusion, we misdiagnosed this tumor as a pilocytic astrocytoma, a more common and benign tumor that commonly occurs in children. Therefore, the clinical identification of ependymoblastomas and other primary brain neoplasms remains challenging.

## 4. Conclusions

This report described an atypical case of infratentorial ependymoblastoma, which contributes to the sparse existing literature on ependymoblastomas. Although several advanced MRI protocols were employed, we misdiagnosed this tumor as a benign pilocytic astrocytoma. Future studies are essential to systematically highlight imaging findings that may be used to distinguish ependymoblastoma from other brain tumors, to enhance the diagnostic and therapeutic workflow, and facilitate the effective and accurate diagnosis of this rare tumor type.
